# Molecular epidemiology of multidrug-resistant organisms on mobile phones: an observational study conducted at a German university hospital

**DOI:** 10.1186/s13756-026-01739-2

**Published:** 2026-04-04

**Authors:** Daniel Hack, Tilman G. Schultze, Volkhard A. J. Kempf, Jon Genuneit, Claudia Reinheimer, Stephan Göttig

**Affiliations:** 1 Goethe University Frankfurt, University Hospital, Institute for Medical Microbiology and Infection Control, Paul-Ehrlich-Str. 20, D-60590 Frankfurt am Main, Germany; 2 Goethe University Frankfurt, University Hospital, University Center for Infectious Diseases (UCI), Frankfurt am Main, Germany; 3 Goethe University Frankfurt, University Hospital, Institute of Public Health, Frankfurt am Main, Germany

**Keywords:** Mobile devices, Cell phones, Multidrug-resistant organisms, MRSA, VRE, Disinfection, Infection prevention, Sixth Moment of Hand Hygiene

## Abstract

**Background:**

Mobile phones are integral to modern clinical workflows and increasingly bridge clinical and private environments. Yet, their role as portable high-touch surfaces in infection prevention remains insufficiently characterized. We hence assessed prevalence and molecular epidemiology of multidrug-resistant organisms (MDRO) on mobile phones used by healthcare workers (HCWs) at a German university hospital and compared them with devices used by non-HCWs.

**Methods:**

In this 30-month cross-sectional study, 232 HCW and 241 non-HCW mobile phones were analyzed to determine MDRO prevalence and overall bacterial count. A subset of devices was examined before and after disinfection with alcohol-based wipes. Whole genome sequencing with subsequent core genome multilocus sequence typing (cgMLST) was applied to identify clonal clusters.

**Results:**

MDRO prevalence was significantly higher on HCW phones compared to non-HCW phones (15.1% vs. 0.4%, *p* < 0.001), with particularly high rates on intensive care unit devices and shared phones (23.4% and 23.0%, respectively). Vancomycin-resistant *Enterococcus faecium*, predominantly the endemic ST117/CT71 clone, and methicillin-resistant *Staphylococcus aureus* were detected on 11.2% and 4.7% of devices, respectively. In contrast, no multidrug-resistant Gram-negatives (MDRGN) were identified, despite contamination with susceptible *Enterobacterales* or nonfermenters. cgMLST analyses revealed clonal MDRO strains mainly within wards and only rarely across wards, consistent with both local clustering and possible cross-ward dissemination. Total bacterial count did not predict MDRO detection. Alcohol-based wipes reliably eliminated MDRO from all tested devices.

**Conclusions:**

Given the high MDRO burden on HCW mobile phones and genomic clustering across wards, mobile phones may represent a relevant reservoir with potential to facilitate in-hospital MDRO dissemination. Standardized mobile phone disinfection routines - particularly for shared and ICU devices - should be considered in infection prevention strategies to reduce potential phone-associated MDRO transmission risk and may represent a conceptual “Sixth Moment” complementing the WHO “Five Moments for Hand Hygiene”.

**Supplementary Information:**

The online version contains supplementary material available at 10.1186/s13756-026-01739-2.

## Background

Mobile phones have become an integral part of daily life and are firmly established in healthcare settings where their use and versatility are expected to increase further [1, 2]. These devices serve diverse functions, ranging from communication and messaging to medical applications, documentation or even simple tasks such as checking the time [1–4]. Studies indicate that 95–99% of healthcare workers (HCWs) use mobile phones for both work-related and personal purposes during their shifts, frequently alternating between patient contact and device handling [1, 2, 5]. Consequently, mobile phones rank among the most frequently touched personal items, with an estimated average of more than 2,500 touches per day [3, 4]. In addition, these devices may serve as fomites harboring diverse bacterial flora, including pathogenic or multidrug-resistant organisms (MDRO) [5–10]. This risk might be amplified when mobile phones, particularly in healthcare environments, are not routinely disinfected. In Germany, to date no specific guidelines or recommendations for mobile phone disinfection have yet been issued neither by authorities nor national infection prevention organizations [11]. Furthermore, the common practice of using private phones for work purposes, and vice versa, reflecting the popular “bring-your-own-device” concept [1–5], potentially increases cross-contamination risks between the healthcare and public sector [2, 7, 12, 13].

In 2021, bacterial antimicrobial resistance was associated with 4.71 million deaths worldwide, including 1.14 million directly attributable to resistant infections [14]. A substantial proportion of these deaths can be linked to five key bacterial pathogens: *Escherichia coli*,* Staphylococcus aureus*,* Enterococcus faecium*,* Klebsiella pneumoniae*, and *Pseudomonas aeruginosa* [14]. Understanding environmental MDRO contamination in healthcare settings, including high-touch portable devices such as mobile phones, may support evidence-based risk assessment and targeted prevention strategies [15–20].

The primary objective of this study was to investigate the prevalence and molecular epidemiology of MDRO on mobile phones used by HCWs at the University Hospital Frankfurt (UMF) in Germany. Findings were compared with MDRO prevalence on mobile phones used by non-healthcare workers (non-HCWs) from UMF. Whole genome sequencing was employed to characterize the molecular features of identified MDRO and to assess phylogenetic relatedness. As a secondary objective, overall bacterial count quantification and non-MDRO contamination rates on mobile phones were investigated, as well as the effectiveness of a standardized disinfection procedure.

## Methods

### Study overview, participants and setting

**Fig. 1:**
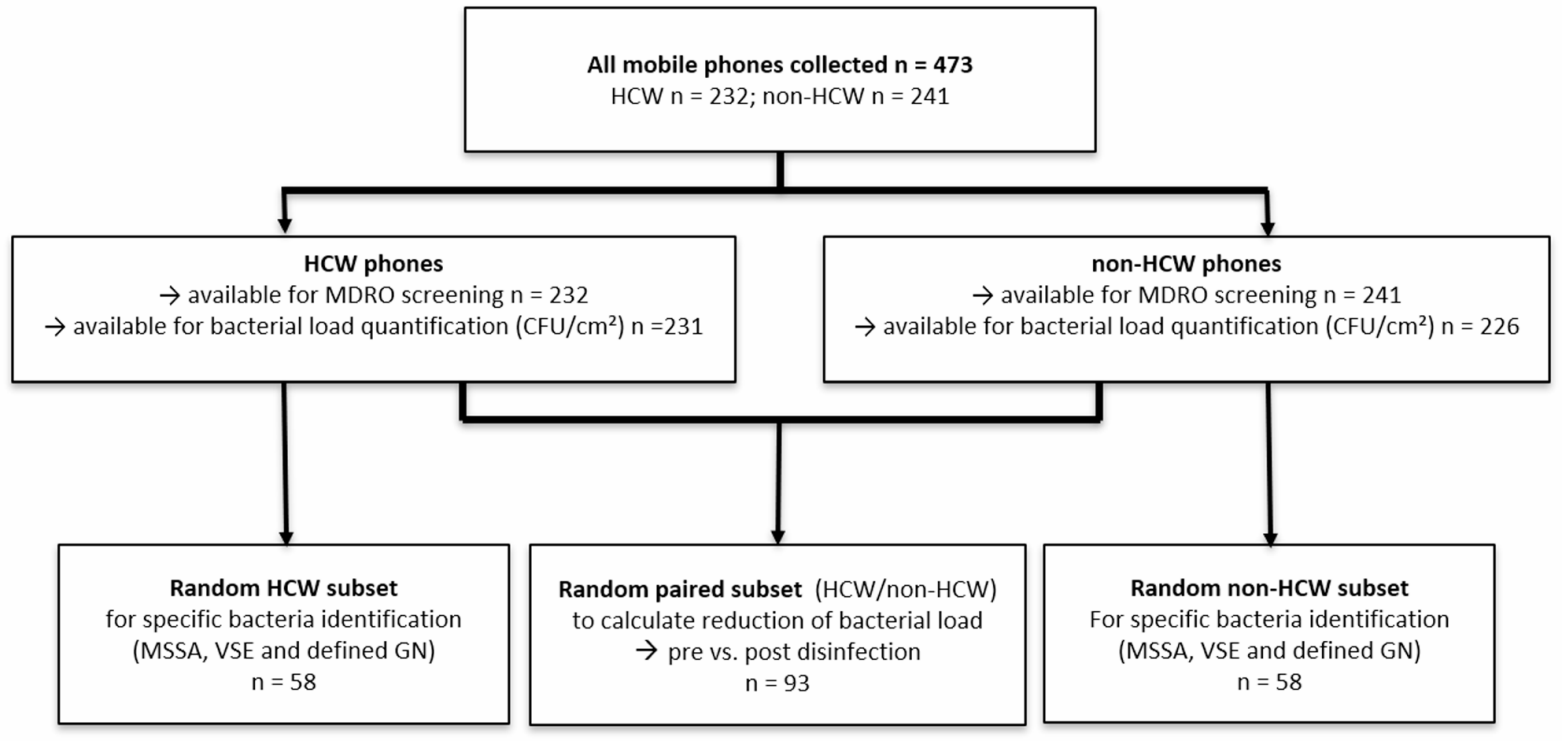
Study overview including design, data availability, workflow and selected subsets. *MSSA* methicillin-sensitive *Staphylococcus aureus*; *VSE*  ancomycin-sensitive enterococci; *GN * Gram-negative bacteria

This study was conducted at the University Hospital Frankfurt (UMF), Germany, representing a tertiary-care center comprising approximately 1,500 patient beds, 33 clinical departments, and more than 11,000 employees and students. At UMF, MDRO patient screening is performed using a risk-based approach (details in the supplementary information). At the time of the study, mobile phone disinfection was not explicitly addressed in the institutional infection prevention and control (IPC) policy, and no mandatory protocol for routine phone disinfection was in place. For the purpose of this cross-sectional observational study, mobile phones of HCWs and non-HCWs were randomly selected over a period of 30 months. Participants were enrolled until the predefined sample size was reached (target ≥ 200 phones per main group); this yielded 232 HCW and 241 non-HCW phones due to ongoing recruitment at the time the threshold was met. All phones underwent MDRO screening as described below; bacterial load was quantified where available. In addition, two subsets were further analyzed: (i) a random subset (*n* = 58 per group) for further characterization of selected clinically relevant pathogens including *S. aureus*, enterococci, *Enterobacterales*, *P. aeruginosa*, and *Acinetobacter* spp. (ESKAPE pathogens), and (ii) a random subset (*n* = 93) for paired pre/post-disinfection assessment immediately before and after alcohol-based disinfection to quantify short-term bacterial reduction and assess residual MDRO detectability. Details of both subset workflows are provided in Fig. 1 and in the supplementary information. Sampling time points and locations were treated as independent observations, and no longitudinal follow-up of individual devices was performed beyond the immediate pre/post assessment. Repeated sampling was not intended. HCWs were defined as individuals with direct patient contact (physicians, nurses, and allied health professionals from different clinical departments and ward types). Non-HCWs were defined as individuals without direct patient contact (medical students in their pre-clinical phase and administrative staff).

Samples from HCW mobile phones were collected during their routine clinical shifts on hospital wards. Samples from non-HCW mobile phones were obtained during work hours or within educational settings. All samples were taken without prior notice. Participation in the study was voluntary. All sampling procedures and subsequent analyses were conducted in accordance with the German Infection Protection Act § 23 [21].

### Sampling, identification and antimicrobial susceptibility testing

Standardized sampling was performed by trained personnel (staff of Institute for Medical Microbiology and Infection Control, UMF) wearing non-sterile disposable gloves to prevent cross-contamination, following careful hand disinfection with an alcohol-based sanitizer. All mobile phones were sampled using 25 cm²-sized RODAC (Replicate Organism Detection and Counting) contact plates containing a disinfectant-neutralizing agent TSA-LTH (Xebios Diagnostics, Düsseldorf, Germany). The contact plate was gently pressed onto the front side of the device, near the “home button” or keypad. Plates were incubated at 36 °C for 48 h, after which colony-forming units (CFU) per 25 cm² were enumerated. Values exceeding the countable range were recorded as > 200 (“overgrown”), treated as right-censored observations and reported separately.

After CFU enumeration, remaining colonies were transferred into CASO broth containing disinfectant-neutralizer LTHTh (Merck, Darmstadt, Germany), vortexed, and processed as described below. Subsequently, because the contact plate covered only part of the device and was primarily used for bacterial load quantification, the entire mobile phone was additionally swabbed with a cotton swab pre-moistened in CASO broth (150 mm; Suesse, Gudensberg, Germany), using a meandering pattern over the front, back, and sides to ensure complete surface coverage. The swab was immersed in CASO broth with disinfectant-neutralizer, vortexed, and incubated at 36 °C for 24 to 48 h. After incubation, 100 µL of each broth was plated onto commercial selective chromogenic agar for MRSA (Brilliance MRSA 2 agar, Thermo Fisher, Darmstadt, Germany), ESBL (CHROMagar ESBL agar, Mast Diagnostica, Reinfeld, Germany), and VRE (ChromID VRE agar, bioMérieux, Nürtingen, Germany), and incubated for 24 h (MRSA, ESBL) or 48 h (VRE) at 36 °C according to the manufacturer’s recommendations. Species identification of suspected MDRO isolates was done using matrix-assisted laser desorption/ionization–time-of-flight (MALDI-TOF) mass spectrometry (bioMérieux). Antimicrobial susceptibility was evaluated by determining the minimum inhibitory concentration (MIC) using the VITEK^®^ 2 system (bioMérieux), with interpretation of MIC according to EUCAST guideline V15.0 [22].

Additionally, random subsets from the two main groups (HCW and non-HCW: *n* = 58 each) were analyzed for clinically relevant pathogens (ESKAPE pathogens) and for overall bacterial growth. This complemented the MDRO screening performed on all devices and enabled comparison of non-MDRO contamination profiles between groups, thereby contextualizing the MDRO findings. Definitions of MDRO and methodical details of the subset analyses are provided in the supplementary information.

As a secondary objective, a randomly selected subset of the HCW and non-HCW devices (overall *n* = 93) was re-sampled post-disinfection procedure using contact plates and swabs, as described above, to quantify bacterial reduction (CFU/25 cm²) and assess residual MDRO presence or survival as described above (Fig. 1). Species identification of suspected non-MDRO after disinfection was done as described in the supplementary information.

### Disinfection of mobile phones

Mobile phones were disinfected using disposable alcohol-based wipes (Bacillol 30 sensitive tissues, Bode Hartmann, Hamburg, Germany) containing 140 mg/g ethanol, 100 mg/g propan-2-ol, and 60 mg/g propan-1-ol, approved explicitly for mobile phone use by the manufacturer [23]. The standardized disinfection procedure was performed by trained personnel and involved wiping the front and back surfaces immediately after removing a fresh wipe from the container. All edges were then wiped to ensure complete surface coverage. If a protective case was present, it was removed and disinfected separately with another fresh wipe. The procedure concluded after a 60-second exposure time, allowing surfaces to air dry.

### Whole genome sequencing and bioinformatic analysis

Whole genome sequencing and bioinformatic analysis were executed from all available MDRO. Unfortunately, two MRSA isolates were not recoverable after cryopreservation and had to be excluded. In short, DNA was extracted from bacterial cultures using a DNeasy UltraClean 96 Kit (Qiagen, Hilden, Germany). Library preparation and sequencing were performed by a commercial service provider (Novogene, Cambridge, United Kingdom) using Illumina two-color sequencing-by-synthesis (SBS) chemistry. Sequencing was performed on a NovaSeq 6000 flow cell using a 2 × 150 bp paired-end sequencing approach. Remaining partial adapter sequences were trimmed from read files using cutadapt version 2.5 and genomes were subsequently assembled using Unicycler v0.4.8. Genome annotation was carried out using Prokka version 1.14.6. Core genome multilocus sequence types (cgMLST) were determined and visualized with Ridom SeqSphere^+^ version 10.5.1 by utilizing cgMLST schemes for *S. aureus* or *E. faecium*, respectively. The cluster threshold for *E. faecium* was set as ≤ 20 alleles as described by de Been et al. [24]. In addition, phylogenetic relatedness was analyzed using the Roary pan-genome pipeline as described [25]. SPA types for *S. aureus* isolates were determined with Ridom SeqSphere^+^. Details are given in Suppl. Table 1.

### Statistical analysis and reporting

Primary statistical analyses were conducted using the software R (R Foundation for Statistical Computing, Vienna, Austria) and SPSS Statistics (version 29.0; IBM Corp., Armonk, NY, USA). To enhance accuracy, 95% confidence intervals were calculated using the Clopper-Pearson method, based on a binomial distribution. For the prevalence of MDRO on mobile phones (= MDRO contamination rate) and subgroup analyses, p-values were determined via Fisher’s Exact test and considered statistically significant at *p* ≤ 0.05 (two-tailed). For aerobic bacterial count quantification, the Mann-Whitney U test was employed for non-normally distributed data and independent groups, with significance set at *p* ≤ 0.05 (two-tailed). The Wilcoxon signed-rank test was used for paired pre/post disinfection measurements, with significance set at *p* ≤ 0.05 (two-tailed). The subgroups were compared for associations and imbalances with Cramér’s V. Adjusted effect estimates were modelled as odds ratios (ORs) using logistic regression under a hypothesized causal model (Suppl. Figure 1). The study is reported in accordance with the STROBE statement for observational studies (STROBE checklist provided in the supplementary information).

## Results

### Prevalence of MDRO and other bacteria on mobile phones

**Fig. 2:**
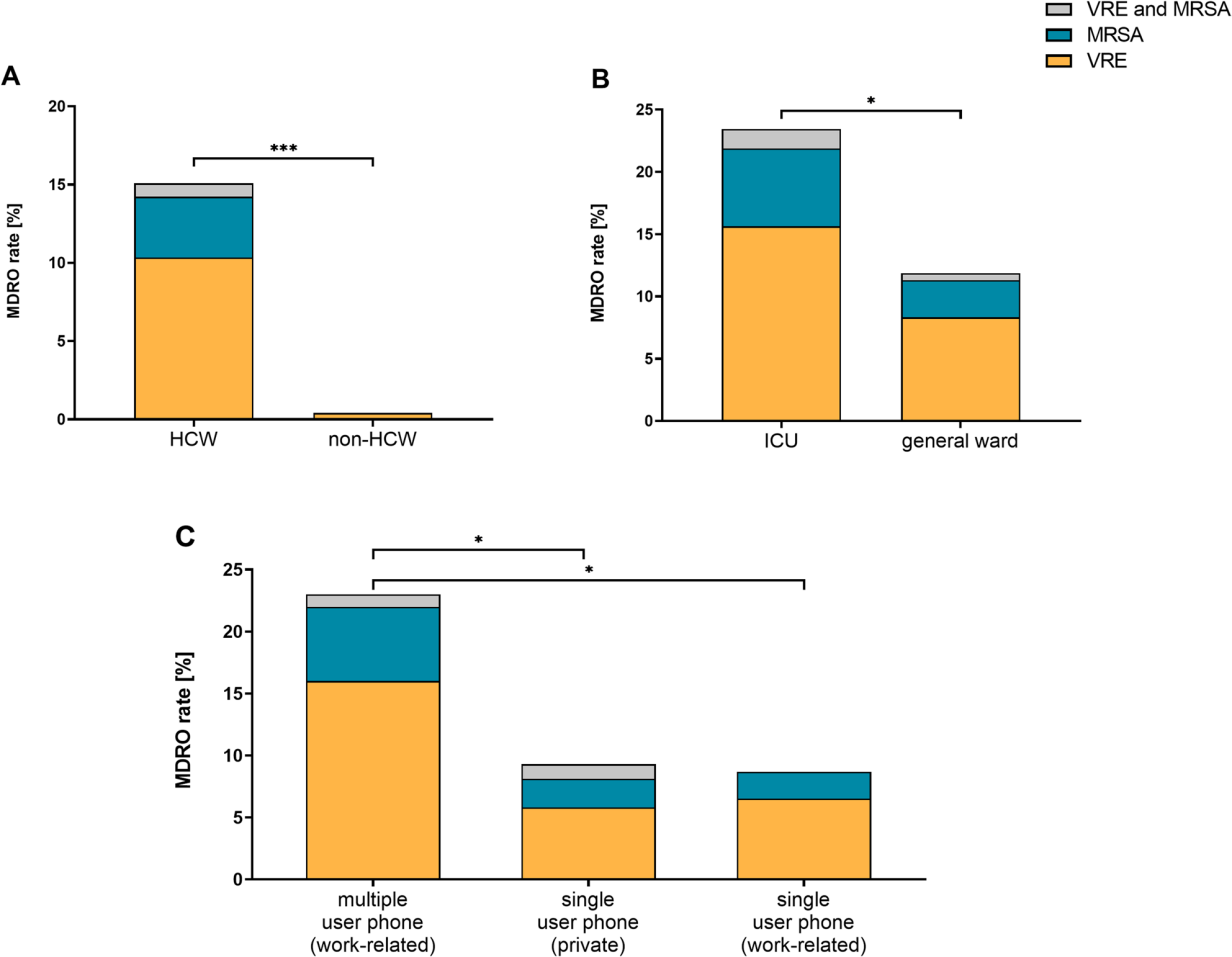
MDRO contamination rates on mobile phones of **A** 232 HCWs and 241 non-HCWs, (**B**, **C**) and in subgroups of HCW. Asterisks indicate statistically significant difference in total MDRO rates. * *p* < 0.05; *** *p* < 0.001 (Fisher’s exact test)

Of 473 mobile phones analyzed in total, MDRO were detected on 36 mobile phones (HCW: *n* = 35/232; non-HCW: *n* = 1/241), resulting in contamination rates of 15.1% on HCW mobile phones and 0.4% on non-HCW devices (Fig. 2A). Only three bacterial species were identified among the MDRO: MRSA, *n* = 11; VRE, *n* = 27 comprising *Enterococcus faecium* (*n* = 26) and *Enterococcus casseliflavus* with intrinsic vancomycin resistance (*n* = 1). No MDRGN were detected on any mobile phones. Two HCW mobile phones were contaminated with two different MDRO (MRSA and VRE) at the same time. Within the HCW group, MDRO prevalence was 4.7% for MRSA and 11.2% for VRE. In contrast, among non-HCW mobile phones, VRE prevalence was 0.4%, with no MRSA detected.

### MDRO contamination in HCW subgroups

MDRO were detected on HCW mobile phones across 15 specialties and in 15 out of 29 wards, most frequently in intensive care units (15/64; VRE *n* = 11, MRSA *n* = 5) and nephrology wards (MDRO *n* = 5/15; all VRE). Consistent with this distribution, subgroup analysis among HCW revealed that mobile phones from ICUs had a significantly higher MDRO contamination rate than those from general wards (15/64, 23.4% vs. 20/168, 11.9%) **(**Fig. 2B). Moreover, work-related mobile phones used by multiple users (e.g. shared devices like on-call, duty or emergency phones) demonstrated a significantly higher MDRO contamination rate (*n* = 23/100; 23.0%) than work-related and private single-user phones (*n* = 12/132; 9.1%) (Fig. 2C). No statistically significant differences in MDRO contamination rates of mobile phones between nurses and physicians or between smartphones and keypad phones were observed (Suppl. Table 2). Potential associations and imbalances between ward type, profession, device type, and phone-use category across HCW subgroups were assessed using cross-tabulations and quantified using Cramér’s V (Suppl. Table 3; Suppl. Figure 2). Nurses were more likely than physicians to use multi-user phones. Smartphones were predominantly private devices, whereas work phones were predominantly keypad phones, leading to sparse or empty cells for several combinations, thereby limiting covariate overlap and precluded disentangling these effects in our study. No difference was observed between private and work single-user phones (Fig. 2C). In unadjusted analyses, multi-user (vs. single-user) phones were associated with higher odds of MDRO contamination (OR 3.14, 95% CI 1.02–9.67). This association strengthened after adjustment for profession (adjusted OR 4.31, 95% CI 1.22–15.28).

### Phylogenetic relatedness of MDRO

**Fig. 3:**
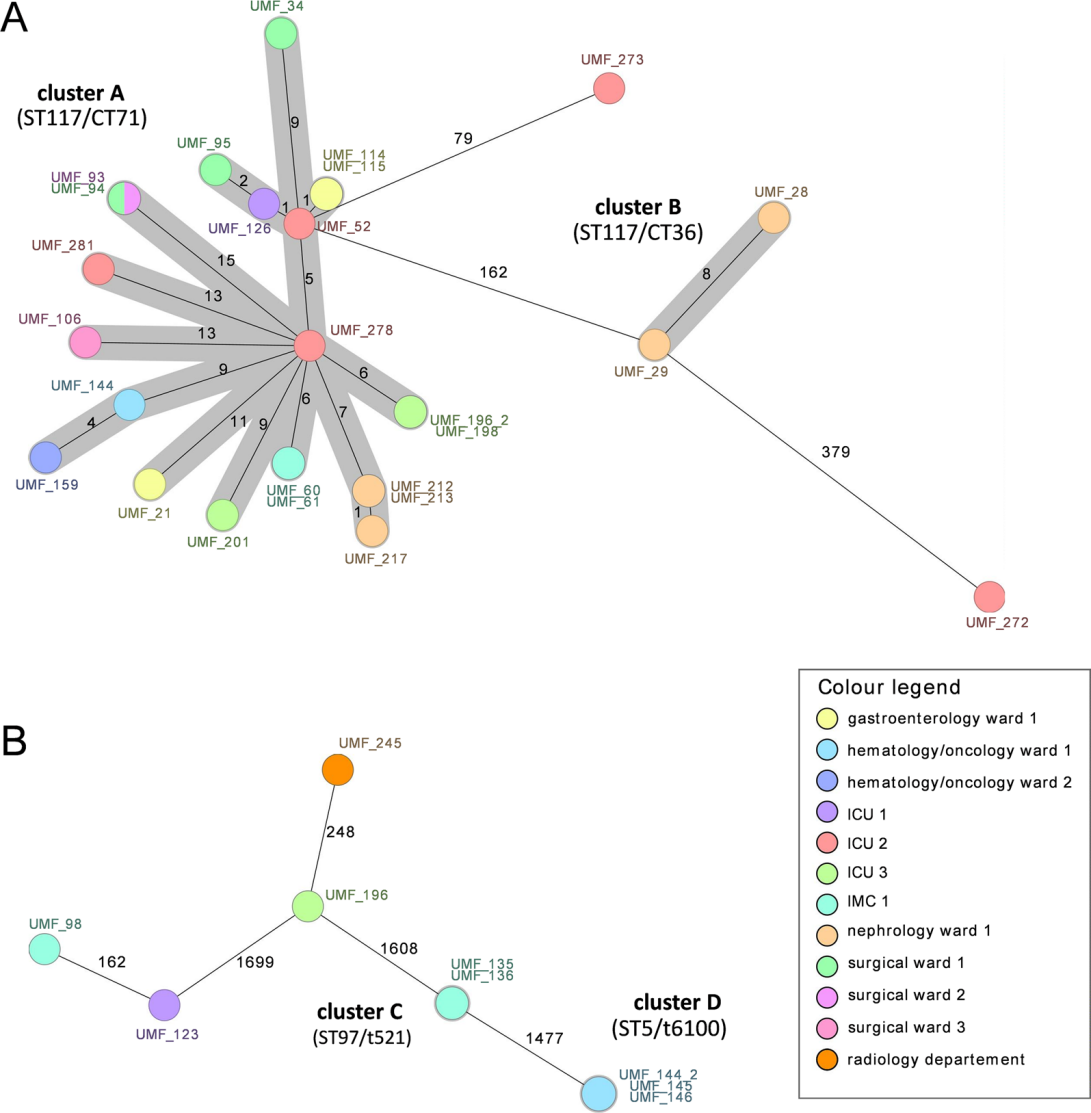
Minimum spanning trees of the 26 VRE (**A**) and nine MRSA (**B**) isolates detected on HCW mobile phones. Each circle represents isolates with an allelic profile based on the sequence of 1,861 core genome targets for *S. aureus* and 1,423 core genome targets for *E. faecium*, as determined by cgMLST analysis. Colours correspond to the origin of the samples. Numbers refer to the allele differences between two isolates

All 26 VRE isolates recovered from HCW mobile phones were whole genome sequenced, and a minimum spanning tree based on cgMLST was calculated to visualize their phylogenetic relatedness (Fig. 3A). Of these, 24 strains belonged to the epidemic sequence type (ST) 117 and the remaining two VRE isolates were assigned to ST80 and ST612. Within the ST117 isolates, two distinct clonal clusters were identified: cluster A, comprising 22 isolates of the cgMLST type CT71 from 14 different hospital wards, and cluster B, consisting of two CT36 isolates collected on the same day from the same nephrology ward.

Whole genome sequencing was also performed on nine MRSA isolates from HCW mobile phones (Fig. 3B). cgMLST-based analysis revealed four different sequence types: three isolates belonged to ST5 and were recovered simultaneously from mobile phones of HCWs from the same hematology ward. Two isolates each were assigned to ST22 (radiology department and ICU 3), ST30 (ICU 1 and IMC 1), and ST97 (IMC 1), respectively. The ST22 and ST30 isolates each belonged to different clusters in cgMLST analysis separated by 248 and 162 allele differences, respectively. In contrast, ST5 and ST97 isolates formed clusters C and D, respectively, with no intra-cluster allele differences, indicating clonality (Fig. 3B). Overall, cgMLST analyses indicate ward-level clustering and the presence of closely related VRE and MRSA lineages across the hospital, without evidence of substantial inter-ward transmission.

### Detection of clinically relevant bacteria species on mobile phones

To assess potential differences in the general bacterial flora, irrespective of antimicrobial resistance, we investigated a randomly selected subgroup of mobile phones belonging to HCWs and non-HCWs (*n* = 58 devices per group, Fig. 1). Among HCWs, *S. aureus* was detected on 22 phones (37.9%; 95% CI 25.5–51.6), enterococci on 17 devices (29.3%; 95% CI 18.1–42.7), and *Enterobacterales* or Gram-negative nonfermenters on three devices (5.2%; 95% CI 1.1–14.4).

Among non-HCWs, *S. aureus* was found on 20 phones (34.5%; 95% CI 22.5–48.1), enterococci on ten devices (17.2%; 95% CI 8.6–29.4), and *Enterobacterales* or Gram-negative nonfermenters on three devices (5.2%; 95% CI 1.1–14.4). On all 116 mobile phones of this subset (HCW and non-HCW) “other bacteria” was reported, consisting most frequently of coagulase-negative staphylococci, micrococci and aerobic spore-forming bacilli. No statistically significant differences were observed between the two groups in terms of general bacterial contamination rates regardless of their antimicrobial resistance status (Suppl. Figure 3). This indicates that the microbiological flora of clinically relevant bacteria found on all mobile phones is broadly similar and dominated by Gram-positive bacteria, consistent with human skin flora.

### Total bacterial count on mobile phones and impact of disinfection

**Fig. 4:**
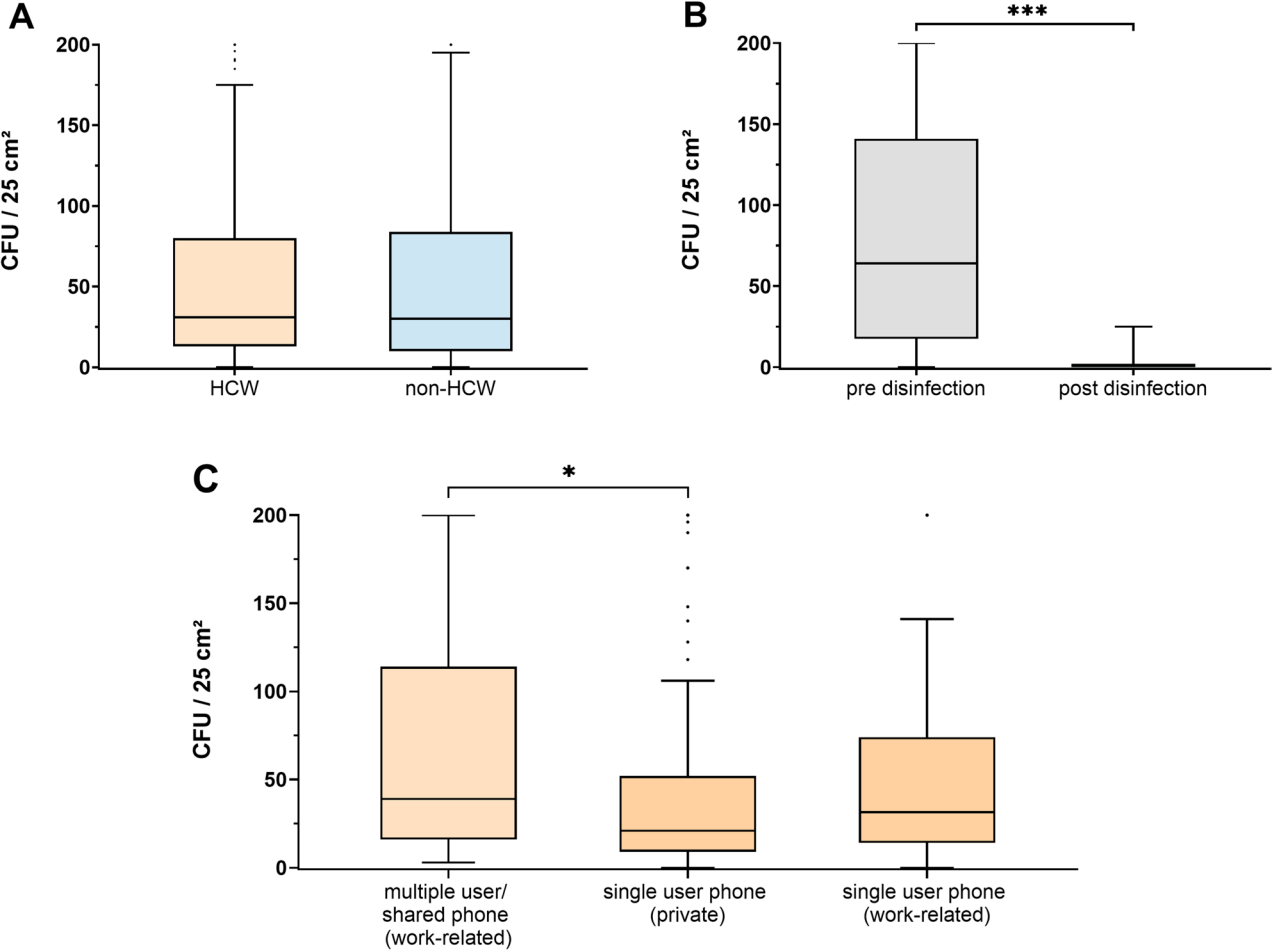
Box-whisker plot (Tukey method) of bacterial counts (as CFU/25 cm²) on mobile phones of HCWs and non-HCWs. Boxes show the interquartile range with the median indicated by a horizontal line. Whiskers extend to values within 1.5x the interquartile range; points beyond represent CFU outliers. **A** Bacterial counts on mobile phones before disinfection in the HCW (*n* = 231) vs. non-HCW group (*n* = 226) and **B** after disinfection with a disposable alcohol-based wipe in a paired subset of 93 randomly chosen mobile phones **C** Bacterial counts on mobile phones according to specific device usage (see figure legend). Statistical significance was determined using Mann-Whitney U test in (**A** and **C**) and Wilcoxon signed-ranked test in (**B**). * indicates significance at *p* < 0.05; *** *p* < 0.001

To quantify the overall microbial burden on mobile phones and assess the impact of disinfection, we compared total aerobic bacterial counts between HCW and non-HCW devices using RODAC contact plates. Mean bacterial counts were similar between groups, with 57.6 ± 61.7 CFU/25 cm² for HCW versus 57.7 ± 63.1 CFU/25 cm² for non-HCW devices (Fig. 4A).

Following disinfection with alcohol-based disposable wipes, the mean bacterial counts in the randomly selected subset were significantly reduced to 1.9 ± 4.2 CFU/25 cm² (Fig. 4B), well below the proposed threshold values for adequately disinfected surfaces (62.5–125 CFU/25 cm²) [11]. The majority of bacteria detected after disinfection were Gram-positive aerobic spore-forming organisms (e.g., *Bacillus* spp., details not shown), which are intrinsically resistant to alcohol-based disinfectants [26]. Notably, in the paired pre/post-disinfection subset, 16.1% (15/93) of devices were MDRO-positive before disinfection, whereas no MDRO were detectable after disinfection.

Among HCWs, no correlation was found between overall aerobic bacterial count and the prevalence of MDRO, which ranged from 8.7% on single-user (work-related) phones to 23.4% on ICU phones (Suppl. Table 2). In the subgroup analysis of HCW mobile phones characteristics, the mean bacterial count was significantly higher on work-related phones with multiple users (70.8 ± 68.3 CFU/25 cm²) compared to phones used by a single individual (work-related and private use: 47.5 ± 54.3 CFU/25 cm²; Fig. 4C). No additional significant differences in the CFU counts were observed regarding profession, ward type, device type or phone-use category (Suppl. Table 2 + 3; Suppl. Figure 2). Total CFU counts were not associated with presence of MDRO (Suppl. Figure 4).

## Discussion

Our study demonstrated that mobile phones, ubiquitous and omnipresent tools in clinical practice [1, 2, 5], can be contaminated with MDRO and may act as potential reservoirs. Although overall bacterial counts on HCW and non-HCW mobile phones were similar (~ 58 CFU/25 cm²), MDRO contamination rates among HCW phones were approximately 37-fold higher (Fig. 2A). MDRO contamination rates were high on both work-related (~ 18%) and personal phones (~ 9%) of HCWs (Fig. 2C). The most frequently detected multidrug-resistant pathogens were VRE, followed by MRSA. This is concerning for two main reasons: (i) mobile phones may serve as reservoirs that could facilitate (multidrug-resistant) pathogen dissemination, thus endangering patients, and (ii) they may contribute to the spillover of hospital-acquired pathogens into HCWs’ private setting and potentially the community [2, 7, 12, 13]. Previously reported MDRO contamination rates between 2 and 15% on mobile phones support our findings [5, 7, 27, 28] although a previous German study reported lower rates of < 2% [5], possibly due to regional differences in MDRO prevalence across Germany [29–32]. MDRGN were not detected on mobile phones in our study, consistent with previous reports indicating reduced environmental persistence of these organisms, especially *Enterobacterales*, compared with Gram-positive bacteria on inanimate surfaces [5, 10, 26]. However, this finding does not exclude MDRGN contamination in other settings, especially where MDRGN prevalence is higher [7, 8]. Notably, two devices yielded more than one MDRO species (e.g., MRSA and VRE). Although these co-detection events were rare, they underscore that individual mobile phones may occasionally accumulate multiple MDRO at once.

Within the HCW cohort, we further stratified phones to explore potential determinants of MDRO contamination and overall bacterial load. CFU counts did not differ between MDRO-contaminated and non-contaminated phones (Suppl. Table 2), and MDRO detection showed no consistent increase across the observed CFU range (Suppl. Figure 4). Together, these observations do not support the intuitive assumption that higher overall bioburden on mobile phones is necessarily associated with a higher MDRO prevalence. One plausible explanation is that total bioburden is strongly influenced by individual patterns, such as cleaning or disinfection practices, rather than by MDRO carriage itself. It is conceivable that some healthcare workers disinfect their phones regularly [5], resulting in low total CFU counts despite intermittent MDRO carriage. Others may rarely clean or disinfect their devices and accumulate higher overall bioburden without necessarily exhibiting higher MDRO rates. From an ecological perspective, a second explanation could be microbial interference or “colonization resistance” [11, 26].

In contrast, MDRO contamination of mobile phones is most plausibly driven by MDRO exposure and prevalence in the clinical environment. Our results show that MDRO contamination rates (MRSA, VRE) aligned with the corresponding patient prevalence: wards with higher MDRO prevalence (e.g. ICUs) showed significantly increased phone MDRO contamination (Fig. 2B) despite comparable overall bioburden (Suppl. Table 2). Shared (multi-user) work-related phones were the only subgroup showing both higher MDRO contamination rates and higher bacterial counts (Figs. 2C and 4C, Suppl. Table 2). This pattern suggests that multiple users increase the probability of MDRO introduction (more potential carriers and more opportunities for transfer) and promote bioburden accumulation through more frequent hand-phone-hand contact [2, 5, 12]. These distinct patterns may support targeted IPC interventions with clearly defined responsibilities, prioritizing high-risk units such as ICUs with especially vulnerable patients and shared devices.

Since 2015, vancomycin-resistant *E. faecium* MLST type ST117/cgMLST complex type CT71 encoding chromosomal *vanB*, has been endemic in the Rhine-Main region, where our hospital is located, and has continued to spread across Germany [29, 30]. As anticipated, the vast majority of VRE from mobile phones in our study belonged to the endemic ST117 (*n* = 24/26), of which 22 belonged to CT71 (Fig. 3A). Furthermore, four VRE isolates representing three different STs (ST117: *n* = 2; ST80: *n* = 1; ST612: *n* = 1) were detected simultaneously on mobile phones of HCWs working on the same ICU. This indicates the concurrent presence of multiple VRE lineages and suggests multiple sources or introductions rather than a single clonal event. Relatedness analysis of MRSA isolates indicated intra-ward clustering involving HCW phones in two instances, potentially facilitated by staff proximity, shared patients, or contaminated surfaces (Fig. 3B). Smartphone sampling, however, captures only a small and selective fraction of the ward microbiota. Thus, the observed genomic relatedness should be interpreted within this limited sampling frame.

Hand-mediated contact is considered the primary transmission route for MDRO in healthcare settings [18–20]. With hand hygiene compliance rates in German hospitals estimated at only 70–80% [33, 34], there is ample opportunity for MDRO transfer to patients, staff, medical devices, and inanimate objects such as mobile phones. While our data cannot quantify or demonstrate MDRO transmission from phones to patients, our findings and prior studies suggest that regular phone disinfection reduces contamination and may mitigate phone-associated MDRO transfer risk [6, 9, 10]. We therefore implemented mobile phone disinfection routines in our local IPC practice. Specifically, we introduced the following disinfection indications: (i) once daily for all phones, (ii) when transferred between wards and/or persons, (iii) after suspected contamination, (iv) at shift changes, and (v) upon leaving the hospital. However, the optimal disinfection frequency cannot be derived from this single time-point study. While such measures may be challenging due to organizational barriers, time pressure, and compliance issues, it is essential that healthcare workers recognize the potential risk of pathogen transmission via mobile phones. A potential strategy is to integrate “mobile phone hygiene” into existing regular hand hygiene training. Conceptually, this could complement the WHO “Five Moments for Hand Hygiene” [35] with a sixth moment emphasizing mobile phone disinfection and hand hygiene before and after device use. Future studies should evaluate the feasibility and effectiveness of such interventions in routine clinical practice.

Our study has some limitations. First, it was designed as a cross-sectional, single time-point assessment without longitudinal follow-up. Therefore, the data characterize contamination at a defined time point but do not allow conclusions on persistence or transmission routes over time. Second, we did not assess regular mobile phone disinfection by participants, which could affect contamination rates. Hand hygiene behavior and compliance were not measured and their association with MDRO contamination of mobile phones cannot be evaluated from our data. Furthermore, the standardized pre/post-disinfection procedure was performed under controlled conditions and may not reflect real-life practice during clinical work. Finally, this was a single-center study in a region with sustained VRE endemicity, which may limit generalizability to other institutions and epidemiological settings.

## Conclusion

Mobile phones are increasingly used both privately and within daily clinical workflows, making them particularly relevant compared with other high-touch surfaces. Approximately 15% of HCW devices harbored MDRO, aligning with ward-level MDRO prevalence in our setting and supporting the notion that mobile phones may serve as reservoirs for VRE and MRSA and potentially contribute to their dissemination. Total bacterial counts did not predict MDRO detection, underscoring that bioburden alone - and its measurement by contact plates, as often used in clinical practice - is an insufficient proxy for MDRO contamination. Based on our findings, we encourage standardized mobile phone disinfection as a precautionary IPC measure, particularly for shared devices and high-risk wards, potentially as a “Sixth Moment” complementing the WHO “Five Moments for Hand Hygiene”.

## Supplementary Information

Below is the link to the electronic supplementary material.

Supplementary Material 1.

## Data Availability

The genome and short-read sequences have been submitted to GenBank under BioProject number PRJNA1413402. The analyzed data supporting the conclusions of this article are included within the article and its supplementary files. Raw data and other datasets used and/or analyzed during the current study are available from the corresponding author on reasonable request.

## Abbreviations

CFU: Colony-forming units
HCW: Healthcare worker
IPC: Infection prevention and control
MALDI-TOF: Matrix-assisted laser desorption/ionization–time-of-flight
MDRO: Multidrug-resistant organisms
MDRGN: Multidrug-resistant Gram-negative bacteria
MRSA: Methicillin-resistant Staphylococcus aureus
VRE: Vancomycin-resistant enterococci
MSSA: Methicillin-sensitive Staphylococcus aureus
VSE: Vancomycin-sensitive enterococci
GN: Gram-negative bacteria
RODAC: Replicate Organism Detection and Counting
ST: Sequence type
CT: Complex type
cgMLST: Core genome multi locus sequence typing
UMF: University Medicine Frankfurt
STROBE: Strengthening the reporting of observational studies in epidemiology
