# Supplementary material for: Molecular epidemiology of multidrug-resistant organisms on mobile phones: an observational study conducted at a German university hospital
Source: Antimicrob Resist Infect Control. 2026 Apr 4;15:52. doi: 10.1186/s13756-026-01739-2 (PMC13067586; doi:10.1186/s13756-026-01739-2)
Supplement: Supplementary file 1 — Supplementary Material 1. [file 13756_2026_1739_MOESM1_ESM.docx]

**Supplementary Information**

**Molecular epidemiology of multidrug resistant organisms on mobile phones: an observational study conducted at a German university hospital**

**Daniel Hack, Tilman G. Schultze, Volkhard Kempf, Jon Genuneit**, **Claudia Reinheimer, Stephan Göttig**

**Supplementary Methods**

**Definition of multidrug-resistant organisms**

Multidrug-resistant organisms (MDRO) were defined as methicillin-resistant S. aureus (MRSA), vancomycin-resistant enterococci (VRE), and multidrug-resistant Gram-negative bacteria (MDRGN) employing clinical breakpoints set by EUCAST V15.0 [22] representing the most relevant MDRO in the German healthcare setting. The MDRGN group included Enterobacterales exhibiting resistance to third-generation cephalosporins, with additional resistance to fluoroquinolones (FQ) and/or carbapenems, as well as Pseudomonas aeruginosa and Acinetobacter baumannii resistant to piperacillin, any third- or fourth-generation cephalosporin, fluoroquinolones and/or carbapenems.

**MDRO patient screening at UMF**

At UMF, patients in intensive care and high-risk units are screened for MDRO upon admission and weekly as described in detail earlier [36]. Additional screening occurs if risk factors such as recent antibiotic use, prior hospitalization, long-term devices, chronic wounds, or previous MDRB colonization are present, following UMF’s infection control guidelines and national recommendations issued by the Commission for Infection Prevention and Hygiene in Healthcare and Nursing (KRINKO). MRSA screening used nasal and throat swabs; MDRGN screening used throat and rectal swabs; VRE screening used rectal swabs. Wounds were analyzed if present; tracheal secretions replaced throat swabs in intubated patients.

All patient specimens are processed under stringent quality-controlled conditions at the Institute for Medical Microbiology and Infection Control, University Hospital Frankfurt, Germany, in compliance with ISO 15189:2007 standards (certificate No. D-PL-13102-01-00). For each new batch of VRE, MRSA, and MDRGN selective agar plates, sensitivity and specificity are quality-controlled by inoculation with reference strains at varying inoculum concentrations.

**Sampling and identification of selected clinically relevant bacteria species on mobile phones**

Random subsets from the two main groups (HCW and non-HCW: n = 58 each) were analyzed for clinically relevant pathogens including *S. aureus*, enterococci, *Enterobacterales* as well as other Gram-negative nonfermenters and any bacterial growth. This screening utilized the incubated suspensions described above, plated on mannitol salt agar, kanamycin esculin azide agar, and Endo agar plates for selective isolation and identification. In addition, the suspension was also plated on Columbia agar plates with 5% sheep blood (all Thermo Fisher, Darmstadt, Germany) to detect unselective bacterial growth. Presumptive *S. aureus* colonies recovered on mannitol salt agar were subjected to catalase (ID-ASE, bioMérieux) and coagulase/clumping factor (Pastorex Staph plus, BioRad, Marnes-la-Coquette, France) testing. Isolates positive in both tests were identified as *S. aureus.* Colonies consistent with *Enterococcus* spp. on kanamycin esculin azide agar were tested for catalase activity; catalase-negative isolates were classified as *Enterococcus* spp. Gram-negative isolates from Endo agar were identified by MALDI-TOF and further subjected to antimicrobial susceptibility testing as described in the main text.

Bacterial growth on Columbia agar was determined as “other bacteria”, consisting mostly of coagulase-negative staphylococci, tested with catalase and coagulase/clumping factor reactions as described in the main text, and aerobe spore forming bacilli identified via phenotypically growth or microscopy. Environmental specimens were processed under stringent quality-controlled conditions at the Institute for Medical Microbiology and Infection Control, UMF, in compliance with ISO 17025:2005 accreditation standards (certificate No. D-PL-13102-01-00).

**Species identification of suspected non-MDRO after disinfection**

Microbial growth of suspected non-MDRO on any agar or contact plates after disinfection was primarily assessed phenotypically, consisting mostly of coagulase-negative staphylococci, which were further tested with catalase and coagulase/clumping factor reactions as described in the main text, and aerobe spore forming bacilli identified via phenotypically growth or microscopy.

**Statistical analysis - treatment of non-countable contact plates**

For distributional analyses, primarily countable values (≤200) were analyzed, and the proportion of overgrown plates was reported separately. Additionally, for descriptive purposes only, a conservative capped mean was calculated by setting “> 200” to 200 CFU to estimate a lower-bound mean for better visualization and inclusion of all data points (**Fig. 4**).

**Suppl. Table 1** Software used for bioinformatic analysis

| **Work step** | **Software and Reference including DOI** |
| --- | --- |
| Trimming of read files | cutadapt version 2.5  <https://doi.org/10.14806/ej.17.1.200> |
| Genome assembly | Unicycler version 0.4.8.  <https://doi.org/10.1371/journal.pcbi.1005595> |
| Genome annotation | Prokka version 1.14.6.  <https://doi.org/10.1093/bioinformatics/btu153> |
| cgMLST for *S. aureus* and *E. faecium* | Ridom SeqSphere^+^ version 10.5.1  <https://pmc.ncbi.nlm.nih.gov/articles/PMC4097726/>  <https://pmc.ncbi.nlm.nih.gov/articles/PMC4652124/> |

**Statistical analysis of HCW subgroups using a causal model**

The distribution of phones used by nurses versus physicians and the proportion of single- versus multi-user devices were similar between ICUs and general wards, making further adjustment unnecessary. Accounting for professional imbalance in the analysis of multi- versus single-user phones strengthened the statistically significant association, suggesting that ward type and device sharing - rather than profession per se - are meaningful determinants of MDRO contamination. The sample size limits the precision of effect estimates.


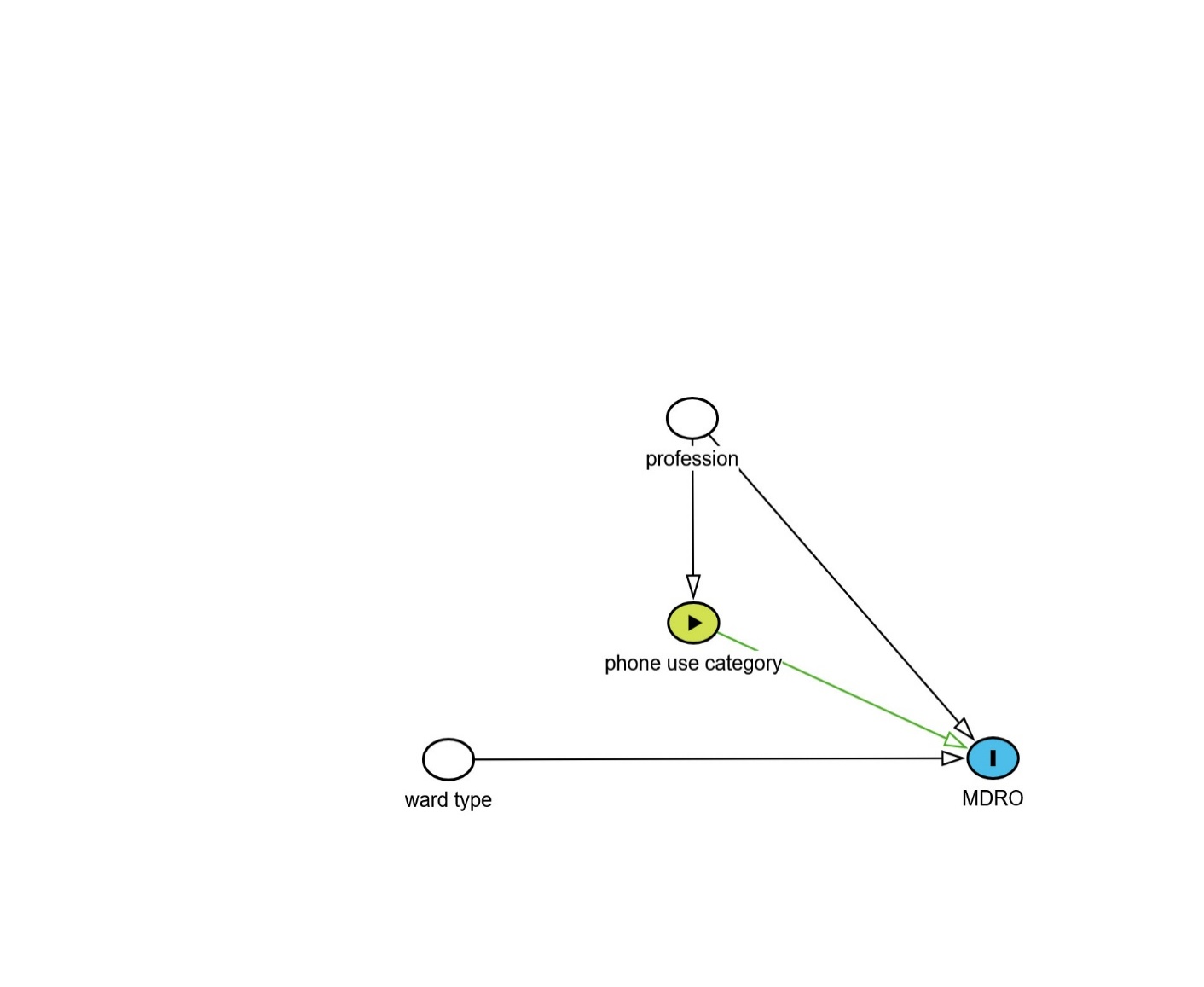


**Suppl. Fig. 1** Causal Model. Ward type has a direct effect on MDRO prevalence on mobile phones, whereas the total effect of profession is partly mediated by phone-use category. Relevant confounding (by profession) occurs only for the effect of phone use category (green) on MDRO prevalence (blue). As in any observational study, unobserved confounding is possible but not depicted in this directed acyclic graph

**Supplementary Results**

**MDRO prevalence in patient screenings at UMF in comparison to MDRO rates on HCW mobile phones**

Across the hospital, the mean MDRO prevalence per 100 patients was 0.45 for MRSA, 1.67 for VRE, and 2.01 for MDRGN. In ICUs, these rates rose to 1.45, 11.41, and 10.81, respectively, indicating a substantially higher MDRO colonization pressure in comparison to the German reference data [32]. Our results show that MDRO contamination rates (MRSA, VRE) correlated with the corresponding patient prevalence: wards with higher MRSA, VRE, and MDRGN prevalence showed significantly increased MDRO contamination rates on mobile phones (see main text). During the study period, MRSA and VRE prevalence in ICU patients treated at UMF were 3.2- and 6.8-fold higher than on non-ICU wards, resulting in only 2.3- and 1.7-fold higher MDRO contamination rates on ICU-HCW mobile phones.

**Suppl. Table 2** Analysis of MDRO contamination rates and mean bacterial count on HCW mobile phones analyzed for subgroups as stated in the table. Asterisks indicate statistically significant difference (*p < 0.05; ** p < 0.001; tests as stated in the table)

| **HCW subgroup** | **MDRO contamination rate in % (n/N)** | **95%-CI (Clopper-Pearson; MDRO)** | **p-value (Fisher-exact-test; MDRO)** | **mean bacterial count ± SD**  **(CFU/25cm^2^)** | **CFU counts > 200 („overgrown“; n)** | **p-value (Mann-Whitney-U-test; CFU/25cm^2^)** |
| --- | --- | --- | --- | --- | --- | --- |
| general ward | 11.9 (20/168) | 7.43 – 17.79 | **0.04*** | 61.7 ± 65.1 | 16 | 0.27 |
| ICU/IMC | 24.2 (15/62) | 14.22 – 36.74 |  | 46.9 ± 50.8 | 2 |  |
| physicians | 14.3 (12/84) | 7.61 – 23.62 | 0.85 | 56.9 ± 62.9 | 7 | 0.53 |
| nurses and others | 15.5 (23/148) | 10.11 – 22.40 |  | 58.0 ± 61.2 | 11 |  |
| smartphone | 9.5 (9/95) | 4.42 – 17.22 | 0.06 | 65.9 ± 72.5 | 12 | 1 |
| key pad phone | 19.0 (26/137) | 12.79 – 26.56 |  | 51.9 ± 52.7 | 6 |  |
| work-related phone (single user) | 8.7 (4/46) | 2.42 – 20.79 | **0.04***  **0.02*** | 53.3 ± 54.1 | 3 | 0.24  **0.002*** |
| work-related phone (multiple users) | 23.0 (23/100) | 15.17 – 32.49 |  | 70.8 ± 68.3 | 12 |  |
| private phone  (single user) | 9.3 (8/86) | 4.10 – 17.51 |  | 44.4 ± 54.5 | 3 |  |
| MDRO-positive | not applicable | not applicable | not applicable | 55.7 ± 59.0 | 3 | 0.61 |
| MDRO-negative | not applicable | not applicable |  | 57.9 ± 62.3 | 15 |  |

**Suppl. Table 3** Association strength / Imbalances between HCW subgroup dimensions using Cramér's V test. Values range from 0 (no association) to 1 (perfect association)

|  | **ward type** | **profession** | **device type** | **phone-use category** |
| --- | --- | --- | --- | --- |
| **ward type** | 1 | 0 | 0.06 | 0.26 |
| **profession** | 0 | 1 | 0.08 | 0.4 |
| **device type** | 0.06 | 0.08 | 1 | 0.78 |
| **phone-use category** | 0.26 | 0.4 | 0.78 | 1 |

| **Cramer‘ V** | 0 | 0 – 0.1 | 0.1 – 0.3 | 0.3 - 0.5 | 0.5 - 1 | 1 |
| --- | --- | --- | --- | --- | --- | --- |
| **Association strength** | none | weak | moderate | Relatively strong | strong | perfect |

**
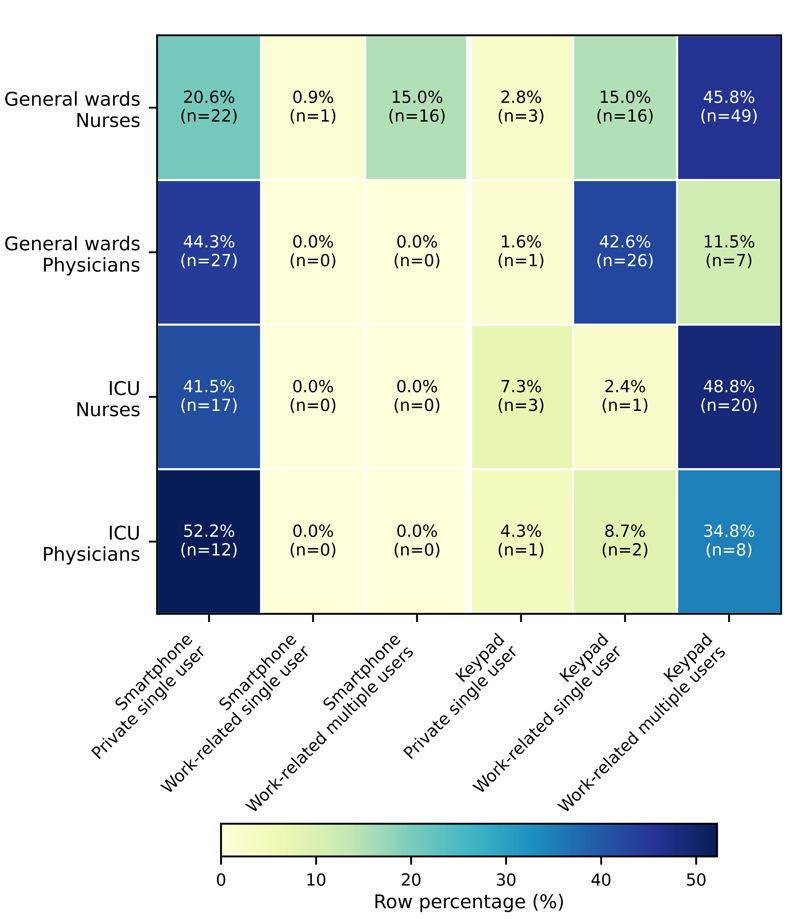
**

**Suppl. Fig. 2 Frequencies and absolute counts of HCW subgroup categories across ward type (ICU vs. general wards), profession (physicians vs. nurses and allied health professions), device type (keypad phone vs. smartphone) and phone-use category (work-related multi user phone vs. work-related single user phone vs. private single user phone)**

**Suppl. Fig. 3** Overall contamination rates and ESKAPE pathogen contamination on mobile phones from 58 HCW and 58 non-HCWs. „Other bacteria“ consisted mainly of coagulase-negative staphylococci and Gram-positive aerobic spore-forming bacilli. Error bars indicate 95% confidence intervals via Clopper-Pearson method

**Association of total CFU count and presence of MDRO on mobile phones**

Bacterial burden and its correlation with presence of MDRO were assessed by plotting a frequency polygon of total viable counts with overlaid absolute MDRO counts (MRSA and VRE) per 5-CFU interval (**Suppl. Fig. 4**). CFU values were non-normally distributed and strongly right skewed: many samples showed very low colony counts, whereas a smaller subset exhibited very high CFU values. Approximately two thirds of all samples had ≤ 50 CFU/25 cm², whereas only about 10% exceeded 200 CFU/25 cm². MDRO were detected across the entire CFU range, and the number of MDRO-positive samples within each interval largely reflected the underlying sample distribution. Overall, there was no statistically significant correlation observed between total CFU counts and presence of MDRO.

**Suppl. Fig. 4 Frequency of CFU values (intervals of five) of HCW phones in comparison to absolute number of MDRO (MRSA and VRE) at each interval. All CFU counts > 200 were defined as “overgrown” and combined in one separate column**

**STROBE Statement** — Checklist of items that should be included in reports of *cross-sectional studies*

|  | Item No | Recommendation | Page No |
| --- | --- | --- | --- |
| **Title and abstract** | 1 | (*a*) Indicate the study’s design with a commonly used term in the title or the abstract | 1 - 2 |
|  |  | (*b*) Provide in the abstract an informative and balanced summary of what was done and what was found | 2 |
| Introduction | | | |
| Background/rationale | 2 | Explain the scientific background and rationale for the investigation being reported | 3 - 4 |
| Objectives | 3 | State specific objectives, including any prespecified hypotheses | 4 |
| Methods | | | |
| Study design | 4 | Present key elements of study design early in the paper | 5 |
| Setting | 5 | Describe the setting, locations, and relevant dates, including periods of recruitment, exposure, follow-up, and data collection | 5 |
| Participants | 6 | (*a*) Give the eligibility criteria, and the sources and methods of selection of participants | 5 |
| Variables | 7 | Clearly define all outcomes, exposures, predictors, potential confounders, and effect modifiers. Give diagnostic criteria, if applicable | 5 - 8 |
| Data sources/ measurement | 8 | For each variable of interest, give sources of data and details of methods of assessment (measurement). Describe comparability of assessment methods if there is more than one group | 6 *- 8* |
| Bias | 9 | Describe any efforts to address potential sources of bias | 5; 16 - 17 |
| Study size | 10 | Explain how the study size was arrived at | 5; 10 |
| Quantitative variables | 11 | Explain how quantitative variables were handled in the analyses. If applicable, describe which groupings were chosen and why | 6 - 9 |
| Statistical methods | 12 | (*a*) Describe all statistical methods, including those used to control for confounding | 8 - 9 |
|  |  | (*b*) Describe any methods used to examine subgroups and interactions | 9 |
|  |  | (*c*) Explain how missing data were addressed | 8 - 9 |
|  |  | (*d*) If applicable, describe analytical methods taking account of sampling strategy | 6 - 8 |
|  |  | (*e*) Describe any sensitivity analyses | 9 |
| Results | | | |
| Participants | 13 | (a) Report numbers of individuals at each stage of study—eg numbers potentially eligible, examined for eligibility, confirmed eligible, included in the study, completing follow-up, and analysed | 5; 10; Fig. 1 |
|  |  | (b) Give reasons for non-participation at each stage | 5; 8 |
|  |  | (c) Consider use of a flow diagram | Fig. 1 |
| Descriptive data | 14 | (a) Give characteristics of study participants (eg demographic, clinical, social) and information on exposures and potential confounders | 10 - 13 |
|  |  | (b) Indicate number of participants with missing data for each variable of interest | 12 - 13 |
| Outcome data | 15 | Report numbers of outcome events or summary measures | 10 - 13 |
| Main results | 16 | (*a*) Give unadjusted estimates and, if applicable, confounder-adjusted estimates and their precision (eg, 95% confidence interval). Make clear which confounders were adjusted for and why they were included | 10 - 13 |
|  |  | (*b*) Report category boundaries when continuous variables were categorized | Not applicable |
|  |  | (*c*) If relevant, consider translating estimates of relative risk into absolute risk for a meaningful time period | Not applicable |
| Other analyses | 17 | Report other analyses done—eg analyses of subgroups and interactions, and sensitivity analyses |  |
| Discussion | | | |
| Key results | 18 | Summarise key results with reference to study objectives | 14 - 15 |
| Limitations | 19 | Discuss limitations of the study, taking into account sources of potential bias or imprecision. Discuss both direction and magnitude of any potential bias | 15 - 17 |
| Interpretation | 20 | Give a cautious overall interpretation of results considering objectives, limitations, multiplicity of analyses, results from similar studies, and other relevant evidence | 16 - 17 |
| Generalisability | 21 | Discuss the generalisability (external validity) of the study results | 16 - 17 |
| Other information | | | |
| Funding | 22 | Give the source of funding and the role of the funders for the present study and, if applicable, for the original study on which the present article is based | 18 |
